# Serological survey of *Coxiella burnetii* at the wildlife–livestock interface in the Eastern Pyrenees, Spain

**DOI:** 10.1186/s13028-016-0209-4

**Published:** 2016-04-27

**Authors:** Xavier Fernández-Aguilar, Óscar Cabezón, Andreu Colom-Cadena, Santiago Lavín, Jorge Ramón López-Olvera

**Affiliations:** 1 Servei d’Ecopatologia de Fauna Salvatge, Departament de Medicina i Cirurgia Animals, Universitat Autònoma de Barcelona, 08193 Bellaterra, Spain; 2 Centre de Recerca en Sanitat Animal (CReSA) - Institut de Recerca i Tecnologia Agroalimentàries (IRTA), Campus de la Universitat Autònoma de Barcelona, 08193 Bellaterra, Barcelona Spain

**Keywords:** *Coxiella burnetii*, Livestock, Pyrenees, Q Fever, Spain, Wildlife

## Abstract

**Background:**

*Coxiella burnetii* is a zoonotic bacterium that infects a wide range of animal species and causes the disease Q fever. Both wild and domestic ruminants may be relevant in the epidemiology of *C. burnetii* infection. In order to investigate the significance of the ruminant host community in the alpine and subalpine ecosystems of the Eastern Pyrenees, Northeastern Spain, in the epidemiology of Q fever, a serological survey was performed on samples from 599 wild and 353 sympatric domestic ruminants.

**Results:**

Specific antibodies against *C. burnetii* were detected with a commercial enzyme-linked immunosorbent assay (ELISA). Domestic sheep showed the highest prevalence (12.7 %, CI 95 % 8.6–16.9), followed by European mouflon (*Ovis orientalis musimon*) with a 6.8 % prevalence (CI 95 % 1.6–12.1), red deer (*Cervus elaphus*) with 2.4 % (CI 95 % 0–5.6), and cattle with a prevalence of 1.1 % (CI 95 % 0–3.2). No positive domestic goats, fallow deer (*Dama dama*), roe deer (*Capreolus capreolus*) and Southern chamois (*Rupicapra pyrenaica*) were detected. Sheep flock prevalence was 75 % (nine of the 12 sheep flocks sampled were positive, within-flock prevalence ranging from 11.1 to 25.0 %), whereas cattle herd prevalence was 11.1 % (one out of the nine cattle herds sampled was positive, within-herd prevalence of 10.0 %.

**Conclusions:**

Both domestic and wild ruminants from the alpine and subalpine ecosystems of the Eastern Pyrenees were exposed to *C. burnetii*. The higher seroprevalence in sheep and its relative abundance suggest that this species may have a major contribution to the ecology of *C. burnetii*. Conversely, wild ruminants do not seem to represent a relevant host community for *C. burnetii* maintenance in the Eastern Pyrenees.

## Findings

*Coxiella burnetii* is a zoonotic bacterium that infects a wide range of animal species and causes the disease Q fever, frequently involving several host species and ticks in natural systems [1–7]. Main sources of human infection are domestic ruminants, which mostly undergo subclinical infections [2]. Wild ruminants may also be relevant in the epidemiology of Q fever, since they can maintain and shed *C. burnetii* [4, 5]. However, the epidemiological role of wild ruminants is unclear and could depend on species features, density and host composition of the ecosystem, and/or the environment [2, 5, 6]. Therefore, research is needed to assess the potential role of wild ruminants in *C. burnetii* epidemiology.

The objectives of this study were to determine the seroprevalence against *C. burnetii* in wild and domestic ruminants in the Eastern Pyrenees, in order to assess the relative importance of the ruminant host species and to evaluate their potential role in the epidemiology of *C. burnetii* in the study area.

**Fig. 1:**
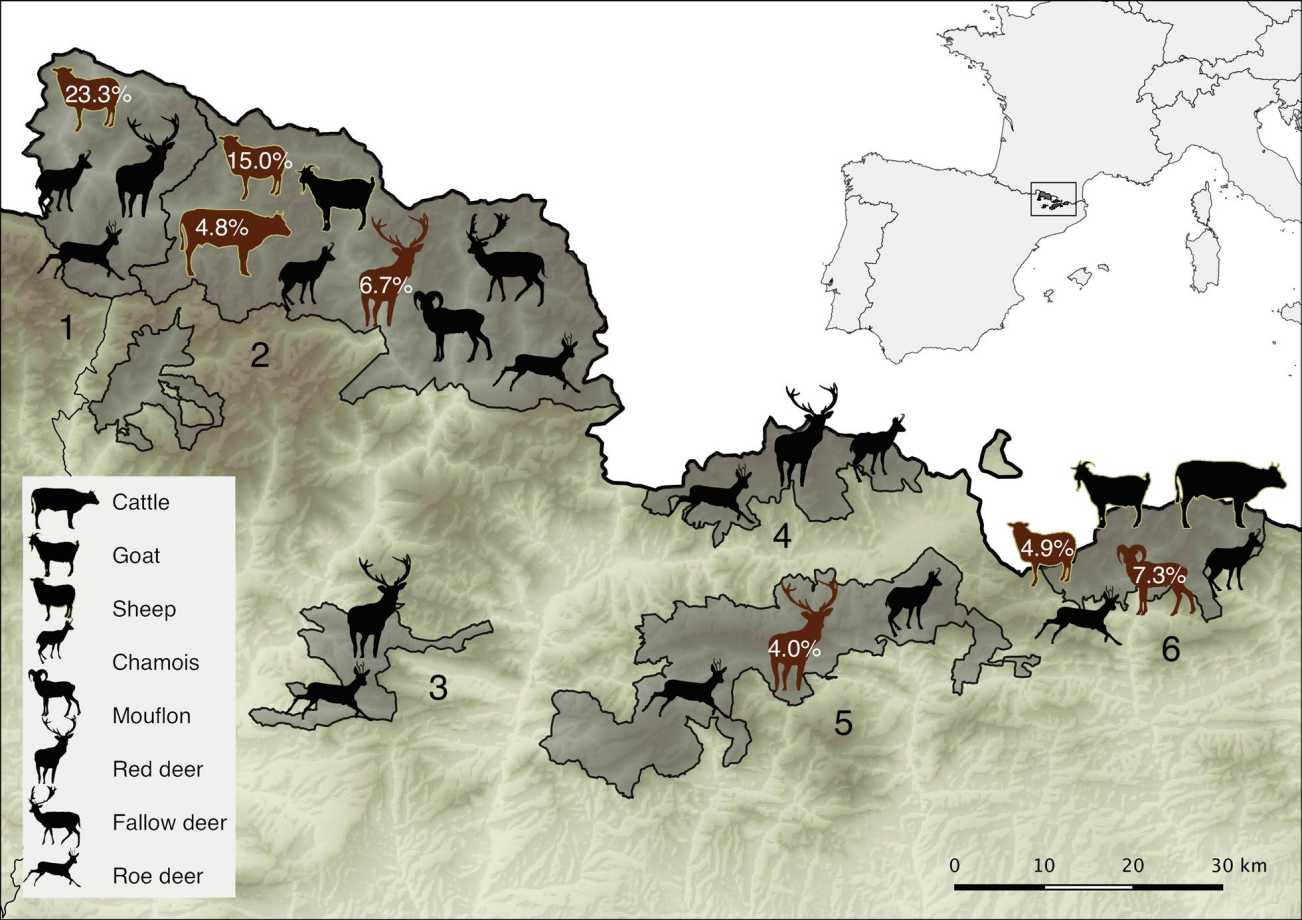
Prevalence of *Coxiella burnetii* specific antibodies assayed by ELISA in the Eastern Pyrenees. Six different management units were sampled: *1* Controlled Hunting Area of Vall d’Aran; *2* National Game Reserve of Alt Pallars; *3* National Game Reserve of Boumort; *4* National Game Reserve of Cerdanya-Alt Urgell; *5* National Game Reserve of Cadí; *6* National Game Reserve of Freser-Setcases

Blood samples from 599 wild and 353 domestic ruminants older than 1 year were collected from 2010 to 2014 in six different management units in the Catalan Eastern Pyrenees, Northeastern Spain (Table 1; Fig. 1). These areas hold most of the wild ungulate population of the Catalan Pyrenees and are managed by the regional administration, which makes them an interesting wildlife–livestock interface scenario and allows reliable sampling and data collection, respectively. These regions are mainly composed by alpine and subalpine ecosystems. Approximately 18,000 wild ungulates dwell in the study areas; 10,000 Southern chamois (*Rupicapra pyrenaica*), 4000 roe deer (*Capreolus capreolus*), 2000 red deer (*Cervus elaphus*), 1000 fallow deer (*Dama dama*) and less than 1000 European mouflon (*Ovis orientalis musimon*) [8, 9]. Approximately 150,000 cattle, 170,000 sheep and 13,000 goats are bred in the corresponding counties [10]. Wild ruminant species were representatively sampled in each study area, since species abundance and composition differs among the management units. Livestock (251 sheep, 11 goats, and 91 beef cattle) were sampled from 21 herds that spend the grazing period from May to November in the Alpine meadows of three of the regions (Table 1; Fig. 1). The sampled herds included 12 sheep flocks (mixed with few goats) and nine beef cattle herds, with herd sizes of 500 and 100 animals, respectively.

**Table 1:** Prevalence of *Coxiella burnetii* antibody positive wild and domestic ruminants in National Game Reserves (NGR) and Controlled Hunting Areas (CHA) in the Eastern Pyrenees, Spain

	NGR Freser-Setcases	NGR Alt Pallars	CHA Vall d’Aran	NGR Cadí	NGR Boumort	NGR Cerdanya-Alt Urgell	Total
Southern chamois (*Rupicapra pyrenaica*)	0/150 0 %	0/36 0 %	0/44 0 %	0/76 0 %	–	0/17 0 %	0/323 0 %
European mouflon (*Ovis orientalis musimon*)	6/82 7.3 % (1.7–13.0)	0/6 0 %	–	–	–	–	6/88 6.8 % (1.6–12.1)
Roe deer (*Capreolus capreolus*)	0/35 0 %	0/16 0 %	0/18 0 %	0/11 0 %	0/1 0 %	0/11 0 %	0/92 0 %
Red deer (*Cervus elaphus*)	–	1/15 6.7 % (0–19.3)	0/6 0 %	1/25 4.0 % (0–11.7)	0/36 0 %	0/3 0 %	2/85 2.4 % (0–5.6)
Fallow deer (*Dama dama*)	–	0/11 0 %	–	–	–	–	0/11 0 %
TOTAL WILD RUMINANTS	6/267 2.2 % (0.5–4.0)	1/84 1.2 % (0–3.5)	0/68 0 %	1/112 0.9 % (0–2.6)	0/37 0 %	0/31 0 %	8/599 1.3 % (0.4–2.3)
Sheep (*Ovis aries*)	4/81 4.9 % (0.2–9.7)	21/140 15.0 % (9.1–20.9)	7/30 23.3 % (8.2–38.5)	–	–	–	32/251 12.7 % (8.6–16.9)
Cattle (*Bos taurus*)	0/70 0 %	1/21 4.8 % (0–13.9)	–	–	–	–	1/91 1.1 % (0–3.2)
Goat (*Capra hircus*)	0/4 0 %	0/7 0 %	–	–	–	–	0/11 0 %
TOTAL DOMESTIC RUMINANTS	4/155 2.6 % (0.1–5.1)	22/168 13.1 % (8.0–18.2)	7/30 23.3 % (8.2–38.5)	–	–	–	33/353 9.3 % (6.3–12.4)
TOTAL	10/422 2.4 % (0.9–3.8)	23/252 9.1 % (5.6–12.7)	7/98 7.1 % (2.0–12.2)	1/112 0.9 % (0–2.6)	0/37 0 %	0/31 0 %	41/952 4.3 % (3.0–5.6)

Positive individuals over the sampled animals are shown, followed by prevalence as a percentage and the CI 95 % between parentheses

The wild ruminant blood samples were obtained directly from the heart from animals hunted during the regular hunting season, mainly from summer to early spring. The livestock samples were obtained from the jugular vein in sheep and goats, and from the medial coccygeal vein in cattle, within the yearly livestock health campaigns. Blood samples were allowed to clot at environmental temperature and transported to the laboratory, where they were centrifuged at 1500×*g* for 10 min. Sera were frozen at −20 °C within 24 h from sample collection and until analysis. Specific antibodies against *C. burnetii* phase I and phase II antigens were tested by a commercial indirect enzyme-linked immunosorbent assay (ELISA) that detects IgG from ruminant species (Q-Fever Antibody Test Kit; IDEXX, Westbrook, Maine, USA). The analyses were performed following the manufacturer’s instructions and results were read at optical density of 450 nm. Although the ELISA used has not been specifically validated for wild species, and both sensitivity and specificity could be lower than those described for domestic ruminants, phylogenetic differences between wild and domestic ruminant species are not higher than among livestock. Moreover, *C. burnetii* ELISA test for livestock have been previously used to study Q fever in wild ruminants [11, 12].

Binomial tests were performed to determine differences between species prevalence, and significance was set at 0.05. All statistical analyses were performed with R software [13]. EpiR package was used to calculate the prevalence estimates [14].

Table 1 shows the seroprevalence estimates for the domestic and wild ruminants. *C. burnetti* ELISA positive individuals were found in European mouflon and red deer and in sheep and cattle. *C. burnetii* antibodies were not detected in domestic goats, Southern chamois, roe deer, and fallow deer. Among the positive species, domestic sheep prevalence was statistically higher than in cattle (p = 0.00255) and red deer (p = 0.01112), but not as compared to mouflon (p = 0.1865). Nine out of the 12 sheep flocks sampled were positive (75 %) and within-flock prevalence ranged from 11.1 to 25.0 %, whereas only one out of the nine cattle herds sampled was positive with a within-herd prevalence of 10.0 %. No sex and age differences were found for *C. burnetii* seroprevalence in any species.

Although previous reports demonstrate that cattle is overall the domestic ruminant species showing a higher overall mean apparent prevalence (20 %) as compared to small ruminants (15 %), sheep and goats may show the highest seroprevalence in certain epidemiological scenarios [5, 6, 15–17]. The higher seroprevalence consistently found in sheep as compared to cattle in the Eastern Pyrenees are in accordance with previous reports in semi-extensive and extensive grazing systems [17]. High *C. burnetii* seroprevalence with zoonotic risk has been reported in goats [12, 13, 18, 19], but the low sample size of our study does not allow to draw strong conclusions. However, the absence of seropositive goats, altogether with the low goat population and the few goats per herd in the study area [10], do not allow to point to this species as important for *C. burnetti* spread in the Eastern Pyrenees.

The seroprevalence against *C. burnetii* found in European mouflon is in accordance with previous reports for this species [20, 21]. Mouflon is taxonomically considered a sheep, and in the Alpine meadows of the National Game Reserve of Freser-Setcases, where *C. burnetii*-seropositive mouflons have been detected, mouflon and sheep commingle and even interbreed. This could explain the high seroprevalence in mouflon being not statistically different from sheep as observed in this study. Red deer has previously been demonstrated as competent hosts for *C. burnetii*, with higher seroprevalences than found in this study. Although red deer has been recognized as an important reservoir host for *C. burnetii* in the Iberian Peninsula, [2, 4, 6], red and roe deer do not seem to play an important role in the ecology of *C. burnetii* in the alpine and subalpine ecosystems of North-Eastern Iberian peninsula. Animal host density and community composition as well as landscape distribution of resources can also influence the dynamics of infectious diseases in wild animal populations [22]. This could explain the different seroprevalences for *C. burnetii* found in studies carried out in different ecosystems with different host communities.

Previous studies have failed to demonstrate antibodies against *C. burnetti* in mountain ungulates such as Alpine ibex (*Capra ibex*) [12] and Southern chamois [23], or has revealed low prevalences in e.g. Alpine chamois (*Rupicapra rupicapra*) [24]. This is in agreement with the findings of our study, where antibodies against *C. burnetii* were not detected in chamois although chamois was the most abundant and sampled wild species in the study areas. Overall, wild ruminant seroprevalence was not related to the species abundance or density. This suggests that other ecological, behavioural and/or environmental factors, and the resulting interspecific contact and transmission rates, may enhance or reduce *C. burnetii* exposure [5, 6].

Inter-species contact between wild and domestic ruminants takes place in mountain habitats [25], which may favour transmission of pathogens such as *C. burnetii*. Wild ruminants can become infected and shed *C. burnetii* as demonstrated for red deer [2, 4, 6] and roe deer [26], and therefore can potentially contribute to the spread of *C. burnetii*. However, the lower seroprevalence found in wild ruminants as compared to livestock suggests that no single species contribute significantly in the maintenance of *C. burnetii* transmission in the alpine and subalpine habitats of the Eastern Pyrenees, but are rather susceptible hosts as a part of the maintenance community in a complex multi-host system [27]. On the other hand, the relatively high overall and within-flock prevalence found in domestic sheep and the consistent detection of high prevalences in sheep in all the studied areas suggest a relative importance of this species as a source of *C. burnetii* spillover events for other susceptible hosts. This is further supported by the fact that sheep is the most abundant species in the study area [10]. This differs from epidemiological scenarios reported elsewhere, where wild ruminant species are recognized as maintenance hosts [2, 4, 6, 7, 26]. Further molecular analyses to characterize the strains infecting sheep and wild ruminants from the Eastern Pyrenees should be performed to ascertain the existence of independent sylvatic and domestic cycles.

To the authors’ knowledge, exposure to *C. burnetii* has been confirmed at the wild-domestic ruminant interface in the Eastern Pyrenees for the first time. This study demonstrates that both domestic and wild ruminants from the alpine and subalpine ecosystems of the Eastern Pyrenees are exposed to *C. burnetii*. The higher seroprevalence in sheep suggests that this species may be of major importance in the ecology of *C. burnetii*. Conversely, wild ruminants do not seem to represent a relevant community of hosts for the maintenance of *C. burnetii*. Identification and characterization of the *C. burnetii* strains infecting domestic and wild ruminants in the Eastern Pyrenees is needed in order to determine whether there is a domestic cycle with spillovers to wild ruminants or independent domestic and sylvatic cycles. Although red deer do not seem to play an important role in the ecology of *C. burnetii*, the finding of positive red deer and European mouflon individuals makes it advisable for hunters and game rangers to use prophylactic measures in order to prevent exposure to *C. burnetii* during post-mortem management of these species.
